# Association Between Shift Work and Acute Coronary Syndrome According to Alcohol Intake and Smoking

**DOI:** 10.3390/medicina61030373

**Published:** 2025-02-21

**Authors:** Seok-Jin Ryu, Sun-Min Kim, Hyun-Yi Kook, Eun-Young Park, Eujene Jung

**Affiliations:** 1 Department of Emergency Medicine, Chonnam National University Hospital, Gwangju 61469, Republic of Korea; samahalak@naver.com; 2 Department of Emergency Medicine, Chonnam National University Medical School, Gwangju 61469, Republic of Korea; 3 Biomedical Research Institute, Chonnam National University Hospital, Gwangju 61469, Republic of Korea; kimsm0003@gmail.com; 4 Department of Nursing, Nambu University, Gwangju 62271, Republic of Korea; 5 Department of Occupational and Environmental Medicine, Chonnam National University Hwasun Hospital, Hwasun 58128, Republic of Korea

**Keywords:** shift work, acute coronary syndrome, alcohol intake, smoking

## Abstract

*Background and Objectives*: Shift work is associated with an increased risk of acute coronary syndrome (ACS) and higher rates of smoking and alcohol consumption. This study examines how smoking and alcohol intake may influence the effect of shift work on ACS risk, indicating a complex interaction among these factors in individuals engaged in shift work. *Materials and Methods*: This investigation utilized data from the Korean Genome and Epidemiology Study (KoGES). Shift work was the primary exposure, and the main outcome was ACS, defined as either myocardial infarction or angina pectoris diagnosed from 2003 to 2020. Cox proportional regression analysis was employed to assess the impact of shift work, smoking, and alcohol intake on ACS incidence. Additionally, we performed an interaction analysis to examine the effects of shift work in conjunction with smoking and alcohol intake on ACS incidence. *Results*: Out of 10,038 participants enrolled during the study period, 3696 (36.8%) met the inclusion criteria. The incidence rate of ACS was 11.88 per 1000 person-years in the shift work group compared to 5.96 per 1000 person-years in the non-shift work group. Using Cox proportional logistic regression, shift work was found to be associated with a hazard ratio (HR) of 1.74 (95% CI, 1.20, 2.53) compared to the non-shift work group. Smoking and alcohol consumption did not exhibit a significant HR for ACS incidence, with HRs of 1.31 (95% CI, 0.98, 1.75) and 0.83 (95% CI, 0.65, 1.07), respectively. In the interaction model, after adjusting for other covariates, shift work was not significantly associated with ACS incidence in current smokers (HR 1.05, 95% CI 0.49, 2.23). However, among non-current smokers, shift work emerged as a significant risk factor for ACS incidence (HR 2.26, 95% CI 1.44, 3.55) (*p* for interaction < 0.01). No interaction was found between alcohol consumption and shift work in relation to ACS incidence. *Conclusions*: Shift work is an independent risk factor for acute coronary syndrome (ACS), particularly among non-current smokers. This finding highlights the need to address both lifestyle and occupational factors when developing strategies to mitigate ACS risk among shift workers. Employers and policymakers should consider implementing targeted workplace interventions to reduce this risk. These may include optimizing shift schedules to minimize circadian disruption, providing regular health screenings focused on cardiovascular health, and promoting healthy lifestyle habits such as balanced nutrition, regular physical activity, and stress management programs. Additionally, workplace wellness initiatives could focus on reducing other modifiable risk factors, such as providing resources for smoking cessation and limiting exposure to occupational stressors. Integrating these strategies into occupational health policies can contribute to the early detection and prevention of ACS, ultimately improving the cardiovascular health of shift workers.

## 1. Introduction

Acute coronary syndrome (ACS) refers to a range of conditions, including ST elevation myocardial infarction (STEMI), non-ST elevation myocardial infarction (NSTEMI), and unstable angina [1,2]. Globally, ACS is a leading cause of morbidity and mortality, significantly impacting public health. In previous reports, ischemic heart disease, which includes ACS, accounted for approximately 9 million deaths worldwide in 2021, reflecting a 15.5% increase over the past decade. Additionally, ACS contributes to a substantial burden of disability, with an estimated 182 million disability-adjusted life years (DALYs) lost annually due to ischemic heart disease [3,4].

ACS not only plays a crucial role in sudden cardiac events but also significantly contributes to the progression of heart failure and its related complications [5,6]. Therefore, effective treatment of ACS, along with reducing its occurrence in populations at higher risk due to various factors, is vital for enhancing public health outcomes [7]. Risk factors for ACS include personal attributes such as age, gender, and obesity status, in addition to family history, lifestyle habits like smoking, alcohol consumption, and physical activity, as well as dietary patterns [8]. Additionally, circadian rhythm disruption, commonly seen in shift workers, contributes to ACS risk through multiple physiological pathways. Disruption of the sleep–wake cycle affects the autonomic nervous system by increasing sympathetic activity and reducing parasympathetic tone, leading to elevated heart rate and blood pressure. It also results in heightened cortisol secretion and altered melatonin production, both of which exacerbate systemic inflammation and oxidative stress. Furthermore, circadian misalignment impairs glucose metabolism and insulin sensitivity, contributing to insulin resistance while also affecting lipid metabolism, leading to dyslipidemia. These combined effects promote endothelial dysfunction, increase arterial stiffness, and elevate the risk of hypertension and atherosclerosis, all of which are critical factors in the development of ACS.

Shift work is recognized as a risk factor for several diseases, including ACS, by disrupting circadian rhythms [9,10,11]. Circadian rhythm disruption caused by shift work affects cardiovascular health through multiple pathways. Night shift work suppresses melatonin secretion, leading to increased oxidative stress and endothelial dysfunction, which contribute to the development of atherosclerosis and coronary artery disease [9,10]. Furthermore, shift work is associated with heightened sympathetic nervous system activity and elevated cortisol levels, resulting in increased blood pressure, insulin resistance, and systemic inflammation—all of which are major risk factors for ACS. Sleep deprivation and fragmented sleep, commonly observed among shift workers, further exacerbate these effects, increasing the long-term cardiovascular burden [11,12]. Previous epidemiological studies have demonstrated that prolonged exposure to shift work significantly elevates the risk of ischemic heart disease and stroke, supporting the hypothesis that circadian misalignment plays a crucial role in cardiovascular pathology [13,14]. Such disruptions lead to numerous adverse health effects, including impaired glucose metabolism, increased insulin resistance, hypertension, dyslipidemia, and, ultimately, the development of coronary artery disease [9]. The intricate relationship between these factors and unhealthy lifestyle choices associated with shift work, such as sleep disorders, smoking, and excessive alcohol consumption, heightens the risk of ACS [10,11,12].

Shift work is also linked to lifestyle modifications, including increased smoking and alcohol consumption, which may further compound the risk of ACS. Several studies have reported that shift workers tend to smoke more frequently than non-shift workers, often as a means to cope with fatigue, maintain alertness, or manage occupational stress [8,15]. Additionally, alcohol consumption is often higher among shift workers due to disrupted social and recreational patterns, increased psychological stress, and reduced sleep quality [16]. While moderate alcohol consumption has been associated with certain cardiovascular benefits, excessive intake can exacerbate hypertension and metabolic syndrome, further increasing the likelihood of ACS among shift workers [17]. These behavioral factors may act as mediators or effect modifiers in the association between shift work and cardiovascular disease risk.

Smoking is a significant modifiable risk factor for ACS, contributing to the development of atherosclerosis and endothelial dysfunction. These conditions facilitate plaque accumulation in the coronary arteries [13,14]. The relationship between alcohol consumption and ACS is complex. While moderate alcohol consumption is associated with potential cardiovascular benefits, such as increased levels of high-density lipoprotein and enhanced vascular function, excessive consumption poses various health risks and may lead to conditions that increase susceptibility to ACS [15,16].

The association of shift work, smoking, and alcohol consumption with the risk of ACS is notable. Moreover, shift work itself is a risk factor for behaviors that increase health risks, including smoking and excessive alcohol use. Shift workers often experience circadian rhythm disruption, increased psychological stress, and altered social environments, which may contribute to unhealthy lifestyle behaviors. Prior studies have suggested that these behaviors not only co-exist with shift work but may also modify its impact on cardiovascular outcomes. Consequently, we hypothesized that within the framework of shift work’s effect on ACS risk, smoking and alcohol consumption might act as modifiable factors that either exacerbate or mitigate this risk. Specifically, we expected that current smokers and heavy alcohol consumers among shift workers would exhibit a disproportionately higher risk of ACS compared to non-smokers and moderate or non-drinkers. Conversely, non-smokers and individuals with low alcohol consumption might demonstrate a relatively attenuated ACS risk despite engaging in shift work. This highlights the need to explore these potential interactions to refine ACS risk stratification and develop targeted prevention strategies for shift workers based on their lifestyle behaviors.

Our study aimed to explore the link between shift work as a risk factor for ACS. We examined how this association is potentially moderated by lifestyle choices, such as smoking and alcohol consumption.

## 2. Materials and Methods

### 2.1. Study Design and Data Sources

This study utilized data from the Korean Genome and Epidemiology Study (KoGES; 6635-302), managed by the National Institute of Health under the Korea Disease Control and Prevention Agency, Republic of Korea. Initiated in 2001, KoGES embarked on two distinct prospective cohort studies situated in different locales: Ansung, a rural district with an approximate population of 176,000 in 2010, and Ansan, an urban region with a populace of nearly 715,000 the same year. These studies focused on Korean men and women aged 40 to 69 years who share the same ethnic background. The detailed methodologies, encompassing participant selection and sampling strategies for these ongoing studies, are detailed in other sources [17]. During 2001–2002, a total of 7129 individuals from Ansung and 10,957 from Ansan were deemed eligible. From these, 5018 participants from Ansung (2239 men and 2779 women) and 5020 from Ansan (2523 men and 2497 women) completed the initial assessments in their respective regions. The present study specifically utilized data collected from January 2001 to December 2020. Subsequent follow-up sessions were held consistently, culminating in the 9th assessment between 2019 and 2020. Interviewers adhered to a standardized protocol, receiving updates on procedures biennially. The evaluations for these cohort members took place at two-year intervals.

### 2.2. Study Population and Definition of ACS

The KoGES data encompassed biennial follow-up evaluations of patient information, starting with the baseline survey in 2001–2002. In this context, ACS was characterized as myocardial infarction or angina pectoris identified during the biennial follow-ups from 2003 to 2020. For this analysis, individuals diagnosed with ACS by a physician or those without a history of smoking and alcohol consumption at the baseline survey were omitted.

### 2.3. Shift Work, Smoking, Alcohol Intake, and Other Risk Factors

Participants completed an interviewer-administered questionnaire. In the 2001–2002 questionnaire, we determined exposure to shift work through the question, “Do you engage in shift work that includes night shifts?”, with respondents answering “yes” if applicable. Assessment of smoking and alcohol consumption was based on the response to “Do you currently smoke or consume alcohol?” Additional demographic data collected included age, gender, marital status, and years of education, along with health-related information such as comorbid conditions (hypertension, diabetes mellitus, and dyslipidemia), body mass index, physical activity levels, and insomnia.

### 2.4. Statistical Analysis

We generated descriptive statistics to summarize the baseline characteristics of participants, stratified by shift work, smoking, and alcohol consumption. Baseline characteristics were compared between participants of the KoGES using the Wilcoxon rank-sum test for continuous variables and the chi-square test for categorical variables. Statistical significance for demographic differences was defined as a *p*-value of <0.01. We calculated the crude 19-year (2001 to 2020) incidence rates of ACS as the number of new cases per 1000 person-years, examining the impact of shift work, smoking, and alcohol consumption. Hazard ratios (HRs) and 95% confidence intervals (CIs) from Cox proportional hazards regression models with fixed covariates estimated the relative risks for the 19-year cumulative incidence of ACS, considering shift work, smoking, and alcohol consumption. We also explored the interaction between shift work and smoking/alcohol consumption regarding ACS risk through interaction analysis and assessed multicollinearity among model covariates. All statistical analyses were conducted using SAS version 9.4 software (SAS Institute Inc., Cary, NC, USA).

## 3. Results

### 3.1. Study Findings on Characteristics

Within the study period, 10,038 participants were enrolled, of which 3696 (36.8%) fulfilled the inclusion criteria and underwent further analysis. The distribution of demographic characteristics among the study population based on shift work is depicted in Table 1. The proportion of participants engaged in shift work was 5.98%. Individuals in the shift work cohort predominantly were males who exhibited a higher prevalence of dyslipidemia, increased alcohol consumption, and current smoking habits. In this group, the occurrence of ACS was higher (14.9% vs. 8.0%); however, this difference did not reach statistical significance (*p* = 0.49).

The characteristics according to smoking status are presented in Table 2, with 19.7% of participants identified as current smokers. This group showed a higher proportion of shift workers (13.3% vs. 4.2%), were predominantly male, younger, had a higher prevalence of hypertension and dyslipidemia, increased alcohol consumption, engaged in less vigorous physical activity, and exhibited lower rates of insomnia compared with non-smokers. No significant difference in total ACS cases was observed based on smoking status (10.4% vs. 7.9%, *p* = 0.07).

The characteristics according to alcohol intake are outlined in Table 3, where 55.8% of participants reported alcohol consumption. The alcohol-consuming group had a higher proportion of shift workers, were predominantly male, more likely to smoke, engaged in more vigorous physical activity, and had lower rates of insomnia. No significant difference in total ACS cases was found based on alcohol consumption.

### 3.2. Main Outcomes

The incidence rate of ACS in the non-shift work group was 5.95 per 1000 person-years, while it was 11.88 per 1000 person-years among those with shift work. A Cox proportional hazards regression analysis was performed to determine the effect of exposure variables on ACS incidence, adjusting for all confounding variables. After adjustment, shift work remained significantly associated with an increased risk of ACS (HR: 1.74, 95% CI: 1.20, 2.53) compared to the non-shift work group. However, smoking and alcohol consumption were not significantly associated with ACS risk, with hazard ratios of 1.31 (0.98, 1.75) and 0.83 (0.65, 1.07), respectively (Table 4).

### 3.3. Interaction Analysis

**Figure 1:**
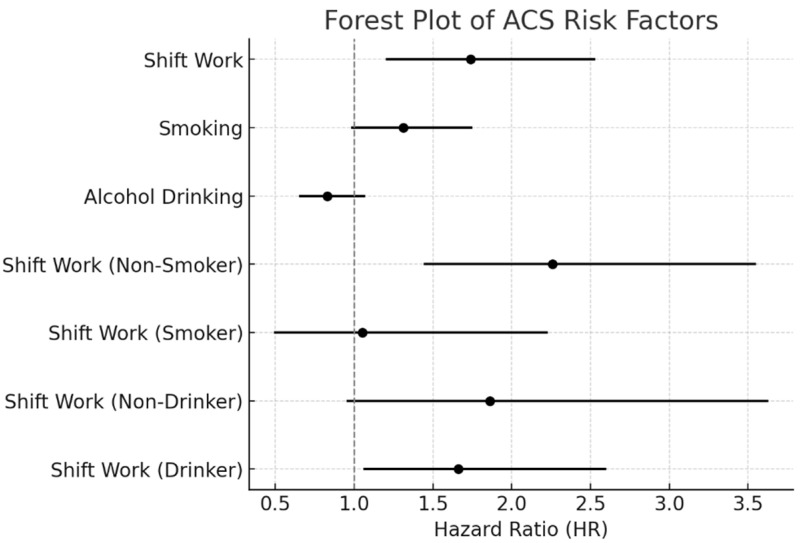
Forest plot of ACS risk factors.

Following the adjustment for additional covariates in the interaction model, the study outcomes’ adjusted hazard ratios (aHRs) varied based on smoking status and alcohol consumption (Table 5) (Figure 1).

The interaction analysis revealed that the association between shift work and acute coronary syndrome (ACS) risk differed significantly by smoking status. Among individuals who currently smoke, shift work was not associated with an increased incidence of ACS (aHR: 1.05, 95% CI: 0.49, 2.23), suggesting no notable effect of shift work in this group. In contrast, among non-smokers, shift work was significantly associated with a higher risk of ACS (aHR: 2.26, 95% CI: 1.44, 3.55), with a statistically significant interaction effect (*p* < 0.01). This finding suggests that shift work may have a stronger adverse impact on ACS risk in non-smokers compared to smokers. However, alcohol consumption did not show a significant interaction with shift work in relation to ACS risk, indicating that the effect of shift work on ACS was not meaningfully modified by alcohol consumption levels.

## 4. Discussion

Our study examined the association between shift work, smoking, alcohol consumption, and the occurrence of ACS. When accounting for potential confounders, we found that shift work significantly increases the risk of ACS compared to individuals not engaged in shift work. Nonetheless, the influence of shift work on ACS incidence was modulated by the individual’s smoking status. For current smokers, shift work did not significantly impact ACS occurrence. In contrast, for those who are not current smokers, shift work was a significant risk factor for developing ACS, highlighting a noteworthy interaction between shift work and smoking status.

Our findings suggest significant correlations between shift work, smoking, and ACS incidence. Notably, shift work poses a risk for ACS, especially among individuals who do not smoke currently. This interaction emphasizes the necessity of considering both occupational and lifestyle factors in cardiovascular risk assessments. Our findings align with previous studies indicating that shift work is a significant risk factor for cardiovascular disease, particularly ischemic heart disease and ACS [8,14]. A longitudinal study conducted among factory workers demonstrated a higher incidence of myocardial infarction among shift workers, supporting the notion that circadian rhythm disruption plays a critical role in cardiovascular risk. However, our study provides a novel insight by highlighting that the effect of shift work on ACS varies based on smoking status. The lack of a significant association between shift work and ACS among current smokers may be attributed to the well-established, dominant impact of smoking on cardiovascular risk, which could overshadow the independent contribution of shift work. This contrasts with findings from prior research, where smoking and shift work were often considered additive risk factors rather than interacting variables. Moreover, our results did not demonstrate a significant interaction between alcohol consumption and ACS risk, diverging from earlier studies suggesting that excessive alcohol intake amplifies cardiovascular risk among shift workers. These discrepancies highlight the need for further research to clarify the potential interactions between occupational and lifestyle factors in ACS development. Consequently, these findings underline the urgency of implementing targeted interventions and health promotion programs in workplaces to mitigate the cardiovascular health risks associated with shift work, especially for non-smokers.

Shift work disrupts the natural alignment of the body’s circadian rhythms, which govern arousal, physiological processes, and behavioral patterns over a 24 h cycle, resulting in shorter and more fragmented sleep during the day [18]. A longitudinal study tracking factory employees over 15 years revealed an elevated risk of ischemic heart disease (IHD) in shift workers compared to day workers, with risk amplifying with prolonged shift work exposure [19]. Furthermore, case–control studies have established a connection between shift work and myocardial infarction [20], alongside evidence linking shift work to an increased risk of atherosclerosis and elevated triglycerides, both known risk factors for ACS [21].

In our study, smoking and alcohol use was elevated among shift workers. It is plausible that some individuals resort to smoking or alcohol as strategies to cope with stress or to maintain alertness during nocturnal shifts [11]. Additionally, the distinct social and lifestyle habits of shift workers, compared to those adhering to standard daytime schedules, may influence the differing rates of smoking and alcohol use [22].

Our interaction analysis revealed no significant effect of the combination of shift work and alcohol consumption on ACS risk factors. However, an interaction was observed with smoking. Among current smokers, shift work did not emerge as a risk factor for ACS. Conversely, for individuals who do not smoke, shift work significantly increases the risk of ACS. This differentiation might be explained by multiple mechanisms. The disturbance of circadian rhythms due to shift work could amplify physiological stress and inflammatory responses, especially noticeable when smoking is not a confounding variable [23]. Furthermore, lifestyle choices associated with shift work, such as erratic eating schedules and diminished physical activity, could heighten ACS risk in non-smokers. Additionally, given smoking’s established effects on oxidative stress and endothelial function, the distinctive risk profile for non-smokers may emphasize the direct influence of shift work on cardiovascular health, distinct from smoking’s harmful effects [24]. From a biological perspective, shift work disrupts the normal oscillations of key circadian genes (e.g., CLOCK, BMAL1, and PER2), leading to dysregulation of metabolic and cardiovascular homeostasis [25,26,27]. This disruption has been associated with increased sympathetic nervous system activation, altered hypothalamic–pituitary–adrenal (HPA) axis function, and subsequent elevations in cortisol and catecholamine levels, all of which contribute to endothelial dysfunction and heightened cardiovascular risk. Moreover, circadian misalignment has been linked to increased insulin resistance, abnormal lipid metabolism, and systemic inflammation, further predisposing non-smokers to ACS. The imbalance of melatonin secretion, a known antioxidant and cardio-protective hormone, may also exacerbate oxidative stress and vascular injury, amplifying the deleterious effects of shift work on cardiovascular health in non-smokers.

Our research is significant as it examines the individual and combined risks of shift work, smoking, and alcohol consumption as potential contributors to ACS. The findings, indicating a heightened risk for non-smokers engaged in shift work, provide a theoretical foundation for developing policies aimed at managing the ACS risk among shift workers. This insight is valuable for devising training and risk management strategies tailored to this workforce.

Our study has several limitations that should be considered when interpreting the results. First, while our research is structured as a prospective cohort study, which allows us to observe the progression of events over time, it lacks the rigor of a randomized controlled trial (RCT) in establishing causal relationships. Although we observed significant associations between shift work and ACS risk, we cannot definitively conclude causality due to the potential influence of unmeasured confounders. Second, despite adjusting for various covariates, the possibility of residual confounding remains. Factors such as genetic predisposition, dietary habits, and occupational stress levels were not measured and could have influenced the observed associations. This may have led to either an overestimation or underestimation of the true relationship between shift work and ACS. Third, the degree of risk associated with shift work may vary depending on the duration and specific nature of the shifts, which our study did not account for. Without detailed information on shift work duration, we could not assess a potential dose–response relationship, possibly affecting the accuracy of our findings. Fourth, the histories of shift work, smoking, and alcohol consumption were self-reported, introducing the potential for recall bias or social desirability bias. Misreporting these behaviors could influence the observed associations. For instance, if shift workers underreported smoking or alcohol consumption, the independent effect of shift work on ACS risk might appear stronger than it truly is. Fifth, we did not separately analyze never-smokers and short-term ex-smokers, which may have influenced the associations observed between smoking status, shift work, and ACS risk. A more detailed stratified analysis by smoking history could provide additional insights into these relationships. Finally, our study focused on a specific population group, which limits the generalizability of our findings. The relationship between shift work, smoking, alcohol consumption, and ACS risk may vary based on ethnicity, socioeconomic status, and access to healthcare. Therefore, caution is warranted when applying our results to other demographic groups with differing lifestyle patterns or occupational environments.

## 5. Conclusions

Shift work emerged as an independent risk factor for ACS, with its impact persisting exclusively among individuals who are not current smokers. This highlights the necessity of integrating lifestyle considerations with occupational risks to reduce the incidence of ACS among shift workers. Workplace health policies should include optimized shift scheduling, regular cardiovascular screenings, and wellness programs promoting physical activity, nutrition, and stress management. Future research should explore the long-term effects of shift work patterns and develop targeted interventions to mitigate cardiovascular risk.

## Figures and Tables

**Table 1:** Characteristics of the study population by shift work status.

Variables	All	Shift Work		
	N (%)	Yes	No	*p*-Value
All	3696 (100.0)	221 (100.0)	3475 (100.0)	
Age, year, mean (SD)	50.4 (7.50)	50.4 (7.51)	50.4 (7.52)	0.14
Gender, female	1772 (47.9)	43 (19.5)	1729 (49.8)	<0.01
Married, yes	3422 (92.6)	203 (91.9)	3219 (92.6)	0.26
Educational period > 9 years	2149 (58.1)	126 (57.0)	2023 (58.2)	0.42
Comorbidity				
Hypertension	422 (11.4)	35 (15.8)	387 (11.1)	<0.01
Diabetes mellitus	189 (5.1)	22 (10.0)	167 (4.8)	0.37
Dyslipidemia	127 (3.4)	16 (7.2)	111 (3.2)	0.01
Body mass index				0.81
Mean, SD	21.7 (8.8)	22.4 (7.5)	21.3 (9.3)	
<18.5 (underweight)	35 (0.9)	2 (0.9)	33 (0.9)	
18.5–24.9 (normal weight)	2026 (54.8)	115 (52.0)	1911 (55.0)	
>25.0 (overweight)	1635 (44.2)	104 (47.1)	1531 (44.1)	
Health-related behavior				
Alcohol intake, yes	2064 (55.8)	169 (76.5)	1895 (54.5)	<0.01
Smoking				<0.01
Current smoker	729 (19.7)	97 (43.9)	632 (18.2)	
Former smoker	68 (1.8)	2 (0.9)	66 (1.9)	
Never smoker	2899 (78.4)	122 (55.2)	2777 (79.9)	
Physical activity, vigorous	1676 (45.3)	103 (46.6)	1573 (45.3)	0.57
Insomnia, yes	1152 (31.2)	77 (34.8)	1075 (30.9)	0.38
Total ACS cases	310 (8.4)	33 (14.9)	277 (8.0)	0.49

SD, standard deviation; ACS, acute coronary syndrome.

**Table 2:** Characteristics of the study population according to smoking status.

Variables	All	Smoking Status		
	N (%)	Current	Former or Non-Smoker	*p*-Value
All	3696 (100.0)	729 (100.0)	2967 (100.0)	
Shift work, yes	221 (6.0)	97 (13.3)	124 (4.2)	<0.01
Age, year, mean (SD)	50.4 (7.5)	48.9 (6.5)	50.8 (7.7)	0.03
Gender, female	1772 (47.9)	30 (4.1)	1742 (58.7)	
Married, yes	3422 (92.6)	698 (95.7)	2724 (91.8)	<0.01
Educational period > 9 years	2149 (58.1)	484 (66.4)	1665 (56.1)	<0.01
Comorbidity				
Hypertension	422 (11.4)	62 (8.5)	360 (12.1)	0.01
Diabetes mellitus	189 (5.1)	47 (6.4)	142 (4.8)	0.06
Dyslipidemia	127 (3.4)	36 (4.9)	91 (3.1)	0.03
Body mass index				0.17
Mean, SD	21.7 (8.8)	21.4 (9.1)	22.0 (6.7)	
<18.5 (underweight)	35 (0.9)	9 (1.2)	26 (0.9)	
18.5–24.9 (normal weight)	2026 (54.8)	414 (56.8)	1612 (54.3)	
>25.0 (overweight)	1635 (44.2)	306 (42.0)	1329 (44.8)	
Health-related behavior				
Alcohol intake, yes	2064 (55.8)	603 (82.7)	1461 (49.2)	<0.01
Physical activity, vigorous	1676 (45.3)	275 (37.7)	1401 (47.2)	<0.01
Insomnia, yes	1152 (31.2)	187 (25.7)	965 (32.5)	<0.01
Total ACS cases	310 (8.4)	76 (10.4)	234 (7.9)	0.07

SD, standard deviation; ACS, acute coronary syndrome.

**Table 3:** Characteristics of the study population according to alcohol intake.

Variables	All	Alcohol Drinking		
	N (%)	Yes	No	*p*-Value
All	3696 (100.0)	2064 (100.0)	1632 (100.0)	
Shift work, yes	221 (6.0)	169 (8.2)	52 (3.2)	<0.01
Age, year, mean (SD)	50.4 (7.5)	49.7 (7.1)	51.5 (7.8)	0.14
Gender, female	1772 (47.9)	612 (29.7)	1160 (71.1)	
Married, yes	3422 (92.6)	1955 (94.7)	1467 (89.9)	<0.01
Educational period > 9 years	2149 (58.1)	1288 (62.4)	861 (52.8)	<0.01
Comorbidity				
Hypertension	422 (11.4)	224 (10.9)	198 (12.1)	0.22
Diabetes mellitus	189 (5.1)	100 (4.8)	89 (5.5)	0.4
Dyslipidemia	127 (3.4)	73 (3.5)	54 (3.3)	0.71
Body mass index				0.77
Mean, SD	21.7 (8.8)	22.1 (7.6)	21.5 (8.1)	
<18.5 (underweight)	35 (0.9)	18 (0.9)	17 (1.0)	
18.5–24.9 (normal weight)	2026 (54.8)	1125 (54.5)	901 (55.2)	
>25.0 (overweight)	1635 (44.2)	921 (44.6)	714 (43.8)	
Health-related behavior				
Current smoking, yes	729 (19.7)	603 (29.2)	126 (7.7)	<0.01
Physical activity, vigorous	1676 (45.3)	980 (47.5)	696 (42.6)	<0.01
Insomnia, yes	1152 (31.2)	585 (28.3)	567 (34.7)	<0.01
Total ACS cases	310 (8.4)	172 (8.3)	138 (8.5)	0.89

N, number; SD, standard deviation; ACS, acute coronary syndrome.

**Table 4:** Cox proportional logistic regression analysis for the study outcome.

Potential Risk Factors	Numbers at Risk	ACS Events	Person-Years	Incidence Rate per 1000 PYS	Model 1	Model 2	Model 3
					aHR (95% CI)	aHR (95% CI)	aHR (95% CI)
Shift work							
No	3475	277	46,576.6	5.95	1.00	1.00	1.00
Yes	221	33	2776.8	11.88	1.91 (1.32, 2.75)	1.77 (1.23, 2.57)	1.74 (1.20, 2.53)
Smoking							
Former or non-smoker	2967	234	40,122.70	5.83	1.00	1.00	1.00
Current smoker	729	76	9230.7	8.23	1.34 (1.01, 1.79)	1.33 (1.00, 1.77)	1.31 (0.98, 1.75)
Alcohol drinking							
No	1632	138	21,752.60	6.34	1.00	1.00	1.00
Yes	2064	172	27,600.90	6.23	0.85 (0.67, 1.09)	0.88 (0.69, 1.13)	0.83 (0.65, 1.07)

ACS, acute coronary syndrome; PYS, person-years; aHR, adjusted hazard ratio; CI, confidence interval; Model 1: Adjusted for age and gender; Model 2: Model 1 plus adjustments for hypertension, diabetes mellitus, dyslipidemia, marital status, and education level; Model 3: Model 2 further adjusted for body mass index, physical activity, and insomnia.

**Table 5:** Interaction analysis between shift work, smoking, and alcohol consumption on the study outcome.

	Shift Work		*p* for Interaction
Current smoker	N0	Yes	<0.01
No	1.00	2.26 (1.44, 3.55)	
Yes	1.00	1.05 (0.49, 2.23)	
Alcohol drinking			0.14
No	1.00	1.86 (0.95, 3.63)	
Yes	1.00	1.66 (1.06, 2.60)	

## Data Availability

The data of this study were obtained from the Korea Centers for Disease Control and Prevention, but restrictions apply to the availability of these data, so they are not publicly available.
